# Study of the immunogenicity of *outer membrane protein A* (*ompA*) gene from *Acinetobacter baumannii* as DNA vaccine candidate *in vivo*

**DOI:** 10.22038/ijbms.2019.30799.7427

**Published:** 2019-06

**Authors:** Hossein Ansari, Maryam Tahmasebi-Birgani, Mahdi Bijanzadeh, Abbas Doosti, Mohammad Kargar

**Affiliations:** 1 Department of Genetics, Marvdasht branch, Islamic Azad University, Marvdasht, Iran; 2 Department of Medical Genetics, School of Medicine, Ahvaz Jundishapur University of Medical Sciences, Ahvaz, Iran; 3 Departments of Biotechnology, Ahvaz Branch, Islamic Azad University, Ahvaz, Iran; 4 Cellular and Molecular Research Center, Ahvaz Jundishapur University of Medical Sciences, Iran; 5 Biotechnology Research Center, Shahrekord Branch, Islamic Azad University, Shahrekord, Iran; 6 Department of Microbiology, Jahrom Branch, Islamic Azad University, Jahrom, Iran

**Keywords:** Acinetobacter baumannii, DNA vaccine, Immunomodulation, In vivo, OmpA Outer membrane – protein, ompA gene

## Abstract

**Objective(s):**

*Acinetobacter baumannii *is one the most dangerous opportunistic pathogens in hospitalized infections. This bacterium is resistant to 90% of commercial antibiotics. Therefore, developing new strategies to cure *A. baumannii*-infections is urgent. The DNA vaccines new approach in which the immunogen can be directly expressed inside the target cells through cloning of immunogen into an expression vector. The outer membrane protein A(OmpA) is one the critical factors in pathogenicity of *A. baumannii* which has been repeatedly described as a powerful immunogen to trigger the immune responses. As the pure form of the OmpA is insoluble, vaccine delivery is very hard.

**Materials and Methods:**

We previously cloned the *ompA* gene from *A. baumannii* into the eukaryotic expression vector pBudCE4.1 and observed that the OmpA protein has been considerably expressed in eukaryotic cell model. In current study, the immunogenic potential of pBudCE4.1-*ompA *has been evaluated in mice model of experimental. The serum levels of IgM, IgG, IL-2, IL-4, IL-12 and INF-γ were measured by enzyme-linked immunosorbent assay (ELISA) after immunization with *ompA*-vaccine. The protective efficiency of the designed-DNA vaccine was evaluated following intranasal administration of mice with toxic dose of *A. baumannii*.

**Results:**

Obtained data showed the elevated levels of IgM, IgG, IL-2, IL-4, IL-12 and INF-γ in serum following the vaccine administration and mice who immunized with recombinant vector were survived more than control group.

**Conclusion:**

These findings indicate ompA-DNA vaccine is potent to trigger humoral and cellular immunity responses although further experiments are needed.

## Introduction

*Acinetobacter baumannii is one the most *clinically relevant bacterium among *Acinetobacter* species which is prevalently common in endemic and epidemic nosocomial infections including pneumonia, bacteremia, urinary tract infection, wound infections and meningitis (1-4). The mortality and morbidity connected to *A. baumannii *has been estimated higher than the other members of *Acinetobacter* species which is partly due* to* multidrug resistant feature of *A. baumannii* into the broad range of antibacterial agents including penicillins, carbapenems, aminoglycosides and cephalosporins(5, 6). This opportunistic bacterium has been recorded as one the most dangerous microbes by Infectious Diseases Society of America (IDSA)(7), so it is not surprising that vaccination against *A. baumannii *is the subject of variety of studies worldwide (8-10). Recently, DNA vaccine approach has been preferred than the previous vaccination strategies regarding to several reasons; DNA vaccine does not contain any weakened or dead pathogenic agents. This is an important advantage when the target organism is non-culturable or cannot be prepared as attenuated form (11, 12). DNA vaccines are engineered expression vectors carrying a desired immunogen sequence to induce both humoral and cellular immune responses against variety of pathogens including bacteria, fungi or viruses (13). Besides, DNA vaccine seems to be safe and produces long lasting immunity (11, 12). By means of reverse vaccinology and proteomics, Moriel *et al. *identified 42 surface-exposed and secreted antigens from *A. baumannii *which could be used as potential vaccine targets. These proteins act as outer membrane proteins (OMPs) (major group), adhesion/haemagglutinins molecules or bacterial enzymes and toxins (14). Targeting of OMPs is recently in attention by researchers as these membrane-bounded porins producing channels to export antibacterial agents outside the cell so produce drug resistant phenotypes (15).The outer membrane protein A (OmpA) is one of the most conserved porins in *A. baumannii* which is actively involved bacterial virulence through biofilm formation, interaction with epithelial cells and induction of apoptosis in them. It can also inactive the complement responses by targeting the factor H in serum (16, 17). Such supporting documents suggest that *ompA* gene can be considered as target sequence to construct a DNA vaccine against *A. baumannii*. We previously cloned *ompA *from *A. baumannii *in eukaryotic expression vector pBudCE4.1 and confirmed that eukaryotic cells subjected to this recombinant construct expressed the OmpA efficiently. To complete our previous study, this project was aimed to investigate the immunization potential of recombinant *pBudCE4.1-ompA* in immunized mice model of experimental by measuring the serum levels of INF-γ, IL-2, IL-4, IL-12, IgG and IgM. We also evaluated the protection value of our recombinant DNA vaccine *in vivo *following the injection of toxic dose of bacteria.

## Materials and Methods

***Experimental animals***

Female BALB/c mice with 16-18 g weight and six-to-eight weeks old were purchased from animal center of Ahvaz Jundishapur University of Medical Science (Ahvaz, Iran). All animals were housed and maintained in a 23 ^°^C room, 50% relative humidityand12 hr light–dark cycle accordance with the Guide for the Care and Use of Laboratory Animals. Mice were allowed to acclimate to the laboratory for 1 week before experiments. All studies and procedures were reviewed and approved by the Institutional Animal Care and Use Committee of Ahvaz Jundishapur University of Medical Sciences. Mice received filtered water and sterilized diet *ad libitum*. Animals were observed daily and clinical signs were noted. Mice were randomly divided to three groups of ten each.

***Immunization design with DNA vaccine***

The DNA vaccine was generated in our laboratory by cloning of *ompA *gene from *A. baumannii* into the eukaryotic expression vector pBudCE4.1. The recombinant construct was termed *pBudCE4.1-ompA* and its function was successfully validated in eukaryotic human dermal fibroblast cells (HDF) model at both levels of RNA expression and protein synthesis (18). Three groups of mice were defined during *invivo* experiments. Group-I, -II and -III were the mice who received the *pBudCE4.1-ompA*, pBu

dCE4.1 vector and phosphate buffer salin (PBS) respectively. For immunization, *pBudCE4.1-ompA* recombinant construct was diluted in sterile saline equal to 0.25 μg/μl of DNA vaccine/injection or an equal volume of Vector and PBS, administered intramuscularly(IM) to the animals every 2 weeks through 4 injections( day 0, day 7, day 14 and day28). After four injections, mice received 100 μg of *pBudCE4.1-ompA* in total. Of note, solutions were made fresh before each administration. One week after each injection (day 0, day 7, day 14 and day 35), blood (0.5 ml) was collected from mouse tail vein into the blood collection tubes, centrifuged at 850 × g for 20 min at 20 ^°^C and the supernatant (serum) was collected and stored at -70 ^°^C until use. 

***Enzyme linked immunosorbent assay (ELISA)***

The total IgM-, IgG-, IL-2-, IL-4-, IL-12- and INF-γ –ELISA (BosterBio, USA) were performed on immunized mouse’s serums collected at the days 0 , 7, 14, 21 and 35 based on manufacturer protocols. 

***Determination of lethal dose (LD***_50_ ***) of A. baumannii***

To calculate the lethal dose (LD_50_) of *A. baumannii*, ten serial dilutions of bacteria (10^5^, 10^6^, 10^7^, 10^8^ and 10^9^ CFU) were prepped. The McFarland standard was used as a reference to adjust the turbidity of bacterial suspensions and calculate the number of bacteria. These dilutions were intranasally injected into the20 mice (4 in each group of bacterial doses). The LD_50_ considered as the lowest concentration of that bacteria causing death in host animal.

***DNA vaccine efficiency challenge***

To examine the immunity potential of the *pBudCE4.1-ompA *as well as its protection efficacy against systemic infection, ten female BALB/c mice six-to-eight week old were intranasally injected with LD_50 _dose of *A. baumannii *following immunization by recombinant construct. Two groups of mice (ten in each) receiving the empty vector and PBS were also considered as control groups. The overall survival of mice was considered in a period of 14 days and compared with each group using Kaplan- Meier estimate. The *P*-value less than 0.05 was considered as statistically significant. 

***Statistical analysis***

Data were presented in Prism® 6 software (GraphPad Software, Inc, La Jolla, CA, USA) and analyzed using one-way ANOVA followed by Newman–Keuls multiple comparison test or Student’s *t*-test. The difference between two groups was considered as statistically significant when the *P-value* was less than 0.05. 

## Results

To evaluate the impact of pBudCE4.1-*ompA* vector on cellular and humoral immune responses, the total serum concentration of IgM and IgG as well as cytokine IL-2, IL-4, IL-12 and INF-γ which has been addressed below.

**Figure 1:**
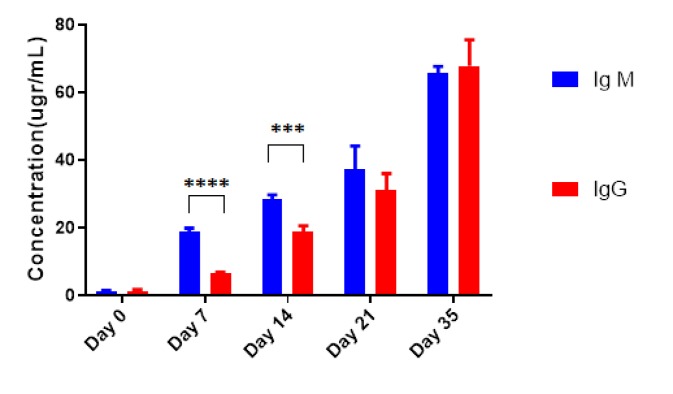
The comparison of IgM and IgG serum levels in mice following administration of 100 μg/μl pBudCE4.1-*ompA* construct. Data were presented as Mean±SD.The *** and **** asterisk indicate the *P*-value less than 10-3 and 10-4 respectively

**Figure 2:**
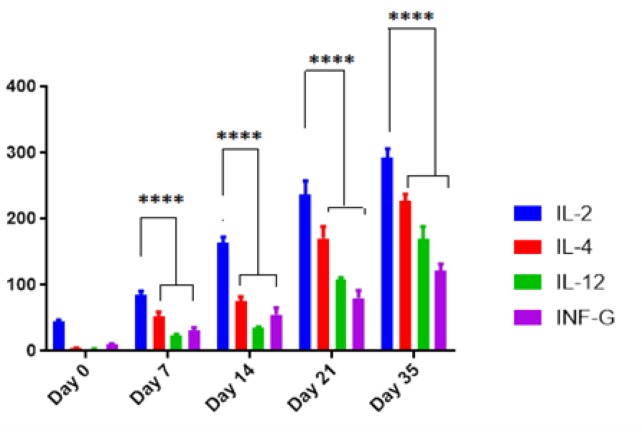
The comparison of IL-2, IL-4, IL-12 and INF-γ serum levels in mice following administration of 100 μg/μl pBudCE4.1-ompA construct. Data were presented as Mean±SD.The **** asterisk indicates the *P*-value less than 10-4

**Figure 3:**
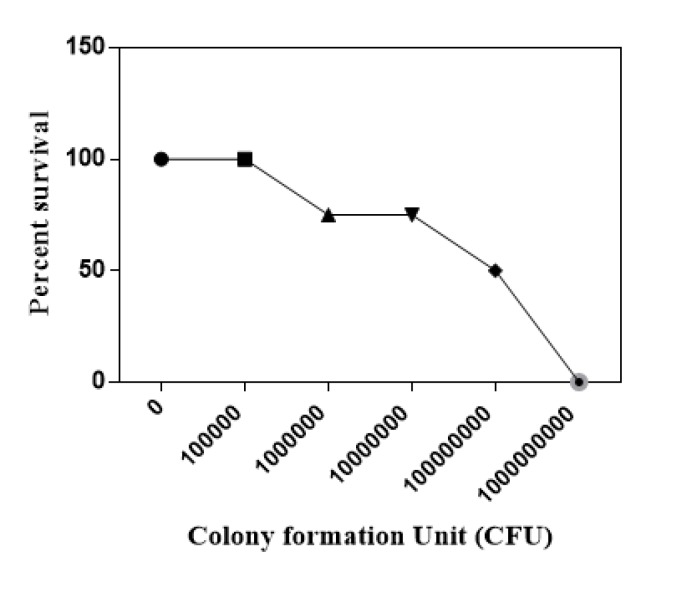
Dose response assay to calculate the median lethal dose (LD_50_) of *Acinetobacter baumannii *in micemodel of experimental

**Figure 4:**
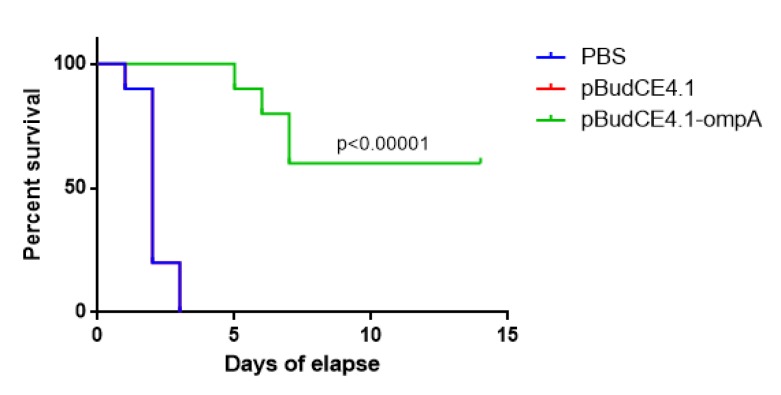
Kaplan-Miere estimate to compare the overall survival among the mice who receive the pBudCE4.1-*ompA*, pBudCE4.1 and phosphate buffer saline (PBS) respectively

**Table1:** The serum levels of IgM and IgG immunoglobulins following theintramuscular injection (IM) of 100μg/μl pBudCE4.1-*ompA*, pBudCE4.1 and PBS. Data presented as Mean±SD

**Immunoglobulin**	**Day 0**	**Day 7**	**Day14**	**Day21**	**Day35**
**pBudCE4.1- ** ***ompA***					
**Ig M**	1.1±0.41	19.13±0.9	28.64±1.2	37.39±6.8	65.8±2
**IgG**	1.3±0.5	6.64±0.38	18.86±1.9	31.36±4.8	67.92±7.75
**pBudCE4.1**					
**Ig M**	1.19±0.4	14.23±0.74	20.9±0.94	23.31±0.76	27.22±0.54
**IgG**	1.3±0.5	4.4±0.5	7.4±1.02	9.8±0.5	14.6±1.2
**PBS**					
**Ig M**	1.19±0.41	4.56±0.53	7.01±0.48	12.9±6.61	13.8±0.9
**IgG**	1.3±0.5	3.20±0.57	4.92±0.46	6.13±0.33	10.5±0.8

**Table 2:** The serum levels of IL-2, IL-4, IL-12 and INF-γ immunoglobulins following the intramuscular injection (IM)of 100μg/μl pBudCE4.1-*ompA*, pBudCE4.1 and PBS. Data presented as Mean±SD

**Immunoglobulin**	**Day 0**	**Day 7**	**Day14**	**Day21**	**Day35**
**pBudCE4.1- ** ***ompA***					
**IL-2**	48±2.5	84.3±6.5	163.7±9.1	237.7±20.1	292.5±13.7
**IL-4**	3.75±1.44	53.1±6.25	75±7.3	170.6±17.8	226.8±10.6
**IL-12**	2.8±0.59	23.15±2.46	35.34±1.56	108±3.125	170.03±18.58
**INF-** **γ**	10.6±096	31.8±4.9	55.25±10.63	80.25±11.47	121.29±10.85
**pBudCE4.1**					
**IL-2**	48.75±3.5	100±15.47	138.75±9.12	162.5±7.5	180±4.78
**IL-4**	3.75±1.44	15.62±3.75	26.8±2.3	38.75±4.3	58.1±3.1
**IL-12**	2.84±0.59	18.31±1.61	24.87±1.3	30.96±2.18	66.59±6.5
**INF-** **γ**	10.6±096	19.4±1.73	26.08±2.09	36.7±2.08	51.29±9.18
**PBS**					
**IL-2**	48.75±3.5	65.62±3.7	93.75±4.08	120±4.78	135±4.7
**IL-4**	3.1±1.25	6.8±2.3	16.8±4.2	26.25±3.2	36.25±3.22
**IL-12**	2.84±0.59	9.09±1.38	19.4±2.24	25.5±0.5	33.6±4.9
**INF-** **γ**	10.6±096	16.5±1.5	24.20±1.04	27.95±2.08	34.6±2.3

**Table 3:** The protection efficacy of DNA vaccine in mice infected with 10^8^ CFU of *A. baumannii* and receiving 100 µg/ µl the pBudCE4.1-*ompA,* the pBudCE4 and phosphate buffer saline (PBS)

**Group**	**IM Injection**	**Challenge**		**% Protection**
		**Live**	**Dead**	
**PBS (N= 10)**	100µl x 4	-	10	(0%)
**pBudCE4.1 (N= 10)**	0.25µg/ µl x 4=(100 µg/ µl)	-	10	(0%)
**pBudCE4.1-ompA (N= 10)**	0.25µg/ µl x 4=(100 µg/ µl)	6	4	(60%)

***The pBudCE4.1-ompA significantly induce the levels of IgM and IgG***

As illustrated in Table 1, following the injection of 100 μg of *pBudCE4.1-ompA*, the level of IgM was significantly induced in a time-dependent manner and increased from 1.1 ±0.4 μg/ml at the day 0 (before immunization) to 65.8±2.16 at day 35 of injection (*P-value*<10^-4^). Although the empty vector or PBS was also increased the level of IgM in a time-dependent manner, these values were significantly less than the corresponding values for mice receiving recombinant construct (*P-value*<10^-4^). The serum level of IgG was also significantly increased following each injections and elevated from 1.3±0.5 at day 0 to 67.92±7.75 at day 35. Similar to IgM, the induction of IgG following the injection of empty vector or PBS were significantly less than the mice receiving pBudCE4.1-*ompA* construct (Table 1). As expected, the IgM was induced and appeared in the serum earlier than the IgG and this difference was remained until day 14 (*P-value*=10^-3^). However, after the third and fourth booster injections, no difference was observed in serum levels of IgM and IgG (*P*-value>0.05) (Figure 1). 

***The pBudCE4.1-ompA significantly induce the levels of IL-2, IL-4, IL-12 and INF-γ***

Table 2, has been summarized the serum levels of IL-2, IL-4, IL-12 and INF-γ following the administration of 100 μg pBudCE4.1-*ompA*, pBudCE4.1 and PBS through four IM injections. The levels of all cytokines increased in a time-dependent manner by increasing the number of booster injections. Meanwhile, IL-2responded to the pBudCE4.1-*ompA* more than the other cytokines (*P-value*<10^-4^) (Figure 2). At the day 35, the serum levels of IL-2, IL-4, IL-12 and INF-γ reached to 292.5±13.7, 226.8±13.7, 170.3±18.25 and 121.29±10.85 pgr/ml respectively which were statistically significant in comparison with corresponding levels in control mice (*P-value*=10^-4^). In fact, the observed immunity by pBudCE4.1 alone and PBS were significantly less than recombinant pBudCE4.1-*ompA *construct (*P-value*=10^-4^). 

***The LD***_50_ ***dose of A. baumanni***

Dose response curve has been prepared following intranasal administration of different doses of bacteria into the mice which is illustrated in Figure 3. The percent of viability of 10^5^, 10^6^, 10^7^, 10^8^ and 10^9^ CFU were equal to %100, %100, %75, %50 and 0, therefore the 10^8^ CFU was considered as the LD_50_ dose of *A. baumanni.*

***The pBudCE4.1-ompA produced 60% protection efficacy in immunized mice***

To calculate the protection efficiency of pBudCE4.1-*ompA*, the lethal dose of *A. baumanni* (equal to 10^8^CFU) was injected to mice after immunization. The mice were considered in a period of 2 weeks. As shown in Figure 4, all the mice who received the vector alone or PBS were dead at the day 2 following the post-injection of lethal dose of bacteria. However, 6 out of 10 from mice subjected to the pBudCE4.1-*ompA* survived until day 14 which was significantly more than two control groups (*P-value*<0.0001). This finding was compatible with the protection efficiency equal to 60% for pBudCE4.1-*ompA *construct.

## Discussion

Here is the first report in which *ompA* gene from *A.baumannii *has been used to construct DNA vaccine against this opportunistic bacteria. *A. baumannii *selected in this study as this bacterium shows multidrug resistant phenotype to 90% of commercial antibiotics which were clinically used to cure infection of these bacteria (19-21). The epidemiologic studies showed rapid emergence of resistant strains of *A. baumannii *in some countries like United State, Italy, Belgium, Korea, Turkey and Iraq so *A.baumannii*-associated infection has been become as a main problem in clinic (19, 22). Similarly, in Iran, the prevalence of multi drug resistant strains in 2001-2007 was estimated equal to 50% which was reached to 74% during 2010-2015. This 24% elevation may partially be due to cross-border migration among countries such as Iraq and Turkey (23, 24). These statistics represent the fact that development of other strategies against *A. baumannii*-infection is urgent and DNA vaccine is in interest these days (14, 25). Among variety of immunogenes in this bacterium, *ompA* gene was considered to develop DNA vaccine in this study due to some reasons. First of all, ompA is conserved among different strains of *Acinetobacter* species. DNA vaccines expressing such conserved antigens are potent and produce the broad-range immunity (26). On the other hand, ompA has stable and soluble structure so it is good target for vaccine design and several studies confirmed that OmpA can induce both humoral and cellular immune responses (27). In a reverse vaccinology approach, Moriel *et al. *identified 42 surface-exposed and secreted antigens from *A. baumannii *that could be used as potential vaccine targets which were cell outer membrane proteins (OMPs) like ompA or proteins with adhesion or haemagglutinins properties (14). Regarding to the above mentioned documents, we have recently cloned *ompA* gene into the eukaryotic expression vector (pBudCE4.1) and transfected it into the eukaryotic cell model and found that the vectors efficiently express the ompA. These observations were confirmed at both RNA and protein levels (18). In the present study, the immunologic properties of pBudCE4.1-*ompA* recombinant have been considered in experimental mice model. Following the injection of *ompA-c*ontaining construct into the mice, the serum level of IgM increased which was significantly higher than the mice injected with empty vector. The IgM is the first appeared antibody in response to initial exposure to an antigen and increased level of IgM in serum is consistent with recent infection in the body (28). Therefore, this finding indicates that the vector successfully express the OmpA protein as an immunogen. Elevated level of IgG was also detected in serum of immunized mice in parallel with IgM although its score was significantly lower than IgM until day 14. 

Following sequential booster injections, the serum levels of IL-2, IL-4, IL-12 and INF-γ increased in immunized mice in time-dependent manner. Among these cytokines, IL-2 allocated the maximum levels in comparison to other above mentioned cytokines. IL-4, IL-12 and INF-γ were abundant in serum after IL-2 respectively. The IL-2 is a protein which can directly interact with its receptors on T lymphocytes and promotes these cells toward differentiation into the effector cells and memory cells (29). The IL-4 can induce differentiation of native Th (Th0) into the Th2 cells. These cells produce IL-4 which can act in a positive feedback loop (30). The stimulatory role of IL-4 on proliferation of B cells and T cells made this cytokine as one the valuable mediator of native and adaptive immunity (30, 31). The IL-4 plays an important role in antibody class switching especially in case of IgE synthesis. The level of IL-12 and INF-γ are also negatively modulated by IL-4 (32). The IL-12 mediates the differentiation of native Th (Th0) into the Th1 cells and naturally produces by macrophage, dendritic cells and neutrophils (33). It can also induce the production of INF- γ and TNF-α from T cells and NK cells and increase the cytotoxicity of these cells (34). The production of IL-12 can also increase by IL-2 (29). The INF- γ is one of the important cytokine in both innate and adaptive immunity and can be synthesis following viral and some bacterial infections (35, 36). This cytokine is naturally synthesized by NK cells, Th1 and CTL cells (37). It can activate macrophages and increase the antigen presentation activity of these cells (38). It can also induce the iNOS enzyme and nitric oxide production to fight with engulfed antigen (38). Regarding to such information, it seems that our designed *ompA*-based DNA vaccine can induce both native and acquired (humoral and cellular) immunity through the production of these four cytokines. Of course, these were the only cytokines that considered in our ELISA assay, so there might be other immune factors to be induced by ompA antigen so further studies are urgent in this way. Previous studies has been also confirmed this fact that *A. baumannii* OmpA protein can trigger innate, humoral and cellular immune responses. As an example, in a study by Lin and coworkers, they found that administration of recombinant OmpA protein with aluminum hydroxide adjuvant increased the mice survival rates through reducing bacterial tissue burdens, and induced high anti-OmpA IgG antibodies titers (39). Two years later, Alzubaidi and Alkozai used the chitosan nanoparticles as adjuvant of OmpA protein to improve the immune response induction and found that IL-2, IL-6, IFN-γ, antibody titer, total leukocytes and differential leukocytes in treated rats significantly increased in comparison with each control group and OmpA group (40). Yen and his colleagues found that recombinant OmpA protein can induce the antibody production through induction of Th1, Th2 and Th17 signaling pathways. It has been evidenced that Th17 signaling plays an important role against bacterial infections (41). In a study by Zhang *et al*. he found that intranasal vaccination with *A. baumannii*-OmpA can induce both systemic and mucosal antibodies (42). It should be emphasized on this point that all the above examples were based on purified or recombinant OmpA. An important problem connected to these methods is the insolubility of OmpA protein following the purification procedures which made the OmpA delivery into the cell very hard (43). In DNA vaccine approach, we overcome to such obstacle as the antigenic *ompA* will be expressed in the cell following the vaccine administration. Challenge with lethal dose of bacteria showed that recombinant construct produced 60% immunity in immunized mice with pBudCE4.1-*ompA *and mice who immunized with the recombinant vector could be survived 14 days after injection of lethal dose of bacteria, however all the mice who received the empty pBudCE4.1vectors or PBS dead at the first days of exposure to the bacteria. This finding presents that efficacy of recombinant vector is acceptable. The same observation has been achieved by Alwaired’s experiments using purified OmpA protein (44). 

These finding showed that *OmpA* gene can be considered for development of *A. baumannii *DNA vaccine although further analysis are needed. As an example, it is necessary to evaluate the *ompA* specific immune activation and calculating anti-ompA titer in the next step. As stated by Lin “ OmpA-based vaccines are very promising as they are highly reproducible, easily to be manufactured commercially, and safer than the whole complex preparations” (39). To improve DNA vaccine efficacy, administration of vaccine with adjutants like nanoparticles of chitosan, (45), dendrosome (46, 47) or other nano-adjuncts are highly recommended.

OmpA become insoluble following purification which makes its delivery very hard (43). Additionally, preparing the bivalent DNA vaccine in which two or more antigens of *A. baumannii* were applied can be promising strategies to power the efficacy of DNA vaccine against *A. baumannii*- associated infections. These are the questions which should be addressed in future studies. 

## Conclusion

Here, we examined the antigenic potential of our recombinant construct pBud4CE4.1-*ompA* in mice model of experimental and found that the serum levels of IgM, IgG, IL-2, IL-4, IL-12 and INF-γ were elevated in mice who immunized with recombinant vector. These mice who received the pBud4CE4.1-ompA survived more than control group following the post-injection with lethal dose of bacteria with protection efficacy of 60%. These observations indicate that *ompA* gene can be considered as promising gene in DNA vaccine expriments although further expriments are needed.
